# Impact of High Risk of Obstructive Sleep-Disordered Breathing on School Performance in Pediatric Age Group: A Cross-Sectional Study

**DOI:** 10.7759/cureus.80358

**Published:** 2025-03-10

**Authors:** Ibrahim Sumaily, Walaa H Algadhi, Alyaj Hakami, Nirmin H Alhazmi, Khalid A Madkhali, Abdulrahman M Yaseen, Maisa A Baiti

**Affiliations:** 1 Department of Otolaryngology, King Fahd Central Hospital, Jazan, SAU; 2 Department of Otolaryngology, Abu-Arish General Hospital, Jazan, SAU; 3 Department of Internal Medicine, Jazan General Hospital, Jazan, SAU; 4 Department of Radiology, King Fahd Central Hospital, Jazan, SAU; 5 Department of Emergency, Samtah General Hospital, Jazan, SAU; 6 Department of Pediatrics, Jazan University, Jazan, SAU; 7 Department of Pediatric Surgery, King Fahad Central Hospital, Jazan, SAU

**Keywords:** academic performance, jazan, paediatric, school, sleep apnoea

## Abstract

Background

We investigated the effects of high risk of obstructive sleep-disordered breathing (OSDB) on school performance in children aged six to 18 years in the Jazan region of Saudi Arabia.

Methodology

In this cross-sectional study, data were collected using an online questionnaire, which included questions on sociodemographic details, pediatric sleep questionnaire-sleep-related breathing disorder (PSQ-SRBD) scale, and academic performance metrics. Statistical analyses were performed to determine the associations between high risk of OSDB and academic achievements.

Results

The study sample comprised 145 (52%) males and 176 (48%) females, with a significant prevalence of OSDB (31.58%). The results indicated a negative correlation between PSQ-SRBD scores and academic grades, with correlation values ranging from -0.28 to -0.369 (p < 0.001). Children with high risk of OSDB were more likely to have lower academic grades, with significant differences in the academic grades between children with and without OSDB. The prevalence of high risk of OSDB was higher in males (63.4% vs. 45.5%; p < 0.001).

Conclusions

OSDB significantly affects the academic performance in children, with a higher incidence in males. A strong correlation was observed between high risk of OSDB severity and lower academic achievement. These findings emphasize the importance of recognizing and addressing the high risk of OSDB in children to improve their educational outcomes.

## Introduction

Obstructive sleep apnea (OSA) is a sleep disorder defined by the American Thoracic Society (ATS) as “a breathing disorder during sleep characterized by prolonged partial upper airway obstruction and/or intermittent complete obstruction (obstructive apnea) that disrupts normal ventilation during sleep and normal sleep patterns” [1]. The prevalence of obstructive sleep apnea syndrome (OSAS) in the pediatric population is approximately 2-4% in Western countries [2]. Although the pathogenesis of adult OSAS has been the subject of numerous studies, several aspects in children still remain unclear. However, several risk factors may contribute to the etiopathogenesis of OSAS, one of the most important risk factors for obesity [3]. Children who are overweight or obese have a higher chance of developing OSAS than those with normal weight [4].

Other risk factors include adenoid and/or tonsil hypertrophy and allergic rhinitis [5,6]. Moreover, craniofacial abnormalities can also cause upper airway obstruction syndrome and some genetic syndromes [7,8]. In addition, moderate-to-severe high risk of obstructive sleep-disordered breathing (OSDB) and snoring also affect the neurocognitive function in children by affecting developing capabilities, as illustrated by cognitive measures in a severity-graded manner. Sleep-disordered breathing (SDB) can adversely impact the academic achievement of children [9].

In 2016, a study published in the USA between October 2006 and October 2014, included 1,010 children aged five to seven years from public schools and broader communities. They found that children’s abilities to achieve academic and adaptive goals may be adversely affected by SDB, which could ultimately hinder their ability to become independent. Their results highlighted the need for SDB awareness, with a focus on children with severe high risk of OSDB [9].

However, no studies have addressed this issue in Jazan. Therefore, we aimed to investigate the impact of high risk of OSDB on school performance among school-aged children in the Jazan region of Saudi Arabia.

## Materials and methods

Study design

This cross-sectional case-control study included pediatric patients with high risk of OSDB in Jazan, Saudi Arabia, a hugely populated region with approximately two million people.

Study tool and data collection

We used an online questionnaire distributed via local social media with a link directing participants to the digital version of the questionnaire, which had three parts (Appendices). The first included questions on socio-demographic data, such as sex, age, educational level, salary, parents’ educational levels, relatives, and chronic illness. The second included questions on the 23 items of the validated Arabic version of the pediatric sleep questionnaire-sleep-related breathing disorder (PSQ-SRBD) scale to assess symptom-complexes associated with snoring, breathing difficulties, mouth breathing, daytime sleepiness, behavioral/inattention, and other symptoms. Each question had three possible responses: yes = 1, no = 0, and do not know = missing. The number of symptom items that receive a positive response (“yes”) is divided by the total number of items that receive a positive or negative response. Thus, the denominator eliminates items with missing responses. The result is a number with a proportion ranging from 0.0 to 1.0. Scores > 0.33 are considered positive and suggestive of a high risk of pediatric sleep-related breathing disorder [10]. The third part of the questionnaire covered academic performance, with five possible responses: excellent, very good, good, pass, and fail.

Sample size calculation

We sought to ascertain the sample size required to accurately estimate the prevalence of high risk of OSDB among the target population. Based on two previous studies, we aimed to detect a prevalence rate of 10-18.5% within the population of interest [11,12]. To ensure statistical rigor, a confidence level of 95% was established a priori along with a 4% margin of error. By employing the normal approximation method to construct confidence intervals (CIs) around a sample proportion, we deduced that a sample size of approximately 362 participants was necessary.

Inclusion criteria

We included Saudi pediatric males and females aged six to 18 years. Data were collected between March and April 2023. 

Ethical approval

Ethical approval was obtained from the Standing Committee for Scientific Research at Jazan University (reference number REC-44/02/297, dated September 15, 2022).

Statistical analysis

Statistical analyses were performed using R, version 4.3 (R Foundation for Statistical Computing, Vienna, Austria). Categorical variables are summarized as counts and percentages. The mean ± standard deviation (SD) and median/interquartile range (IQR) were used to summarize continuous normal and non-normal variables, respectively. The chi-square test of independence was used to assess the associations between categorical variables. Spearman’s correlation coefficient was used to assess whether the grades were significantly associated with sociodemographic characteristics and comorbidities. Ordinal logistic regression was used to assess the factors associated with the odds of higher grades. Hypothesis testing was performed at the 5% significance level.

## Results

The study included data from 367 children (191 (52%) males and 176 (48%) females). One-quarter of the respondents were in upper elementary school (97, 26.4%) and more than one-third were in primary elementary school (145, 39.5%). More than two-thirds of the parents had at least a bachelor’s degree. The average monthly income was 10,000-20,000 SAR (Table 1).

**Table 1:** Descriptive statistics for the study sample Data were summarized using counts and percentages.

	N = 367
Age (years (±SD))	10.8 (3.47)
Sex	
Male (N (%))	191 (52.0%)
Female (N (%))	176 (48.0%)
School level	
Primary elementary	145 (39.5%)
Upper elementary	97 (26.4%)
Intermediate	81 (22.1%)
Secondary	44 (12.0%)
Father education level	
Illiterate	8 (2.18%)
General education	80 (21.8%)
Bachelor’s degree	251 (68.4%)
Master/PhD	28 (7.63%)
Mother education level	
Illiterate	28 (7.63%)
General education	103 (28.1%)
Bachelor’s degree	224 (61.0%)
Master/PhD	12 (3.27%)
Monthly income	
<10,000 SAR	88 (24.0%)
10,000-20,000 SAR	174 (47.4%)
20,000-40,000 SAR	74 (20.2%)
>40,000 SAR	31 (8.45%)

Several comorbidities were reported in the children, including asthma (63, 17.2%) and cardiac problems (6, 1.63%). However, no chronic diseases, including hypertension or diabetes mellitus, were reported in 292 (79.6%) of the children. The total daily and night sleeping time was >9 hours in 205 (55.9%) and 105 (28.6%) of the included children, respectively. Two-thirds of the parents reported that their children slept after 10 PM. The prevalence of high risk of OSDB in the study sample was 31.58% (95% CI: 36.51-41.67). The median PSQ-SRBD questionnaire was 0.23 (IQR 0.09; 0.45) (Table 2).

**Table 2:** Comorbidities and sleep-related characteristics of the respondents Data were summarized using counts and percentages.

Variable	N (%)
Bronchial asthma	
No	304 (82.8%)
Yes	63 (17.2%)
Cardiac	
No	361 (98.4%)
Yes	6 (1.63%)
Other chronic diseases	
No	355 (96.7%)
Yes	12 (3.27%)
No chronic disease	
No	75 (20.4%)
Yes	292 (79.6%)
Total day + night sleeping hours	
<9 hours	162 (44.1%)
>9 hours	205 (55.9%)
Total night sleeping hours	
<9 hours	262 (71.4%)
>9 hours	105 (28.6%)
Child sleeping time	
Before 10 PM	118 (32.2%)
After 10 PM	249 (67.8%)

**Figure 1:**
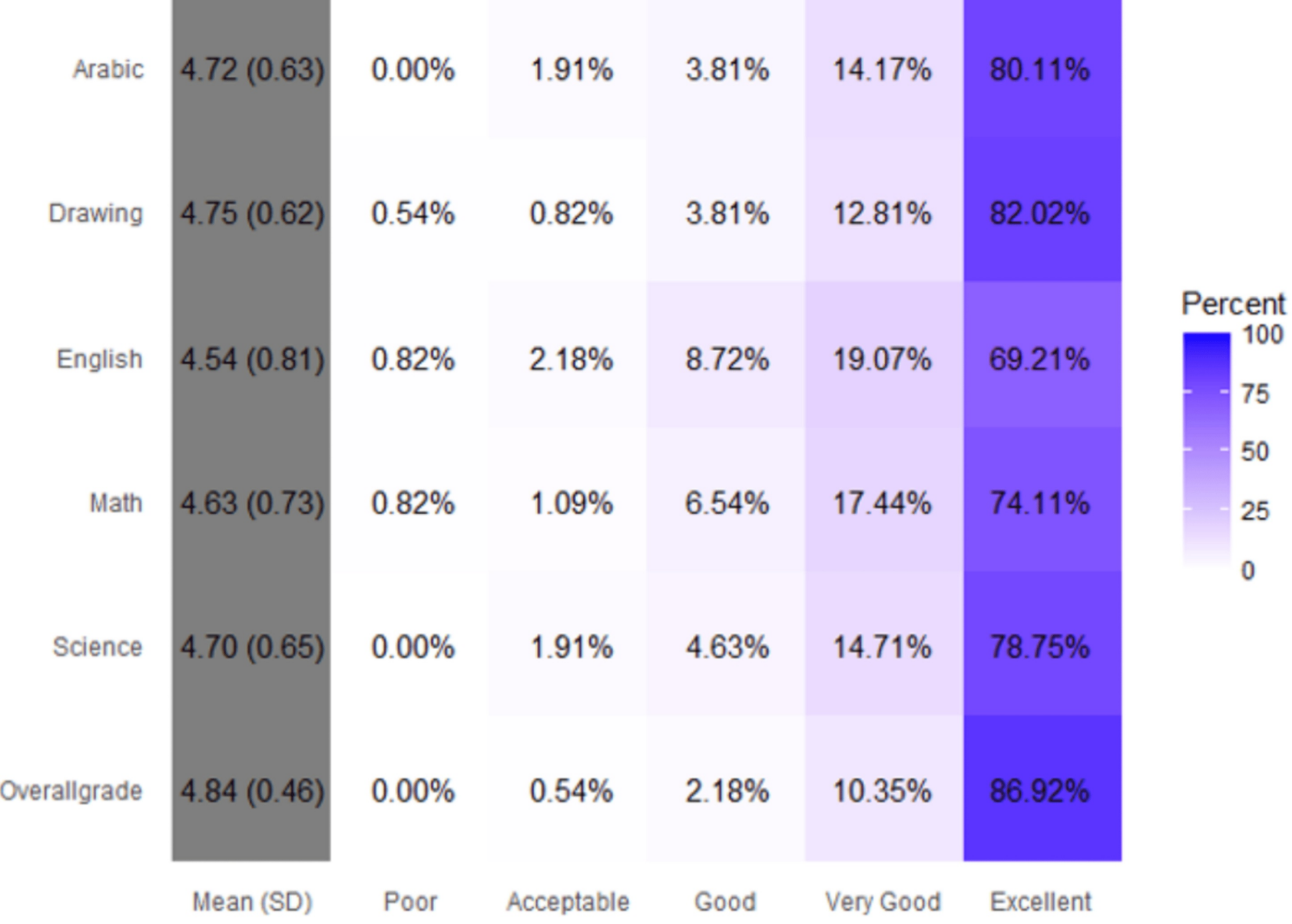
Grades of the included respondents The proportion of children who achieved each grade as well as the average score, assuming excellent = 5 and poor = 1.

More than three-quarters of the respondents achieved excellent grades in all the included subjects and in the overall grade (Figure 1).

**Figure 2:**
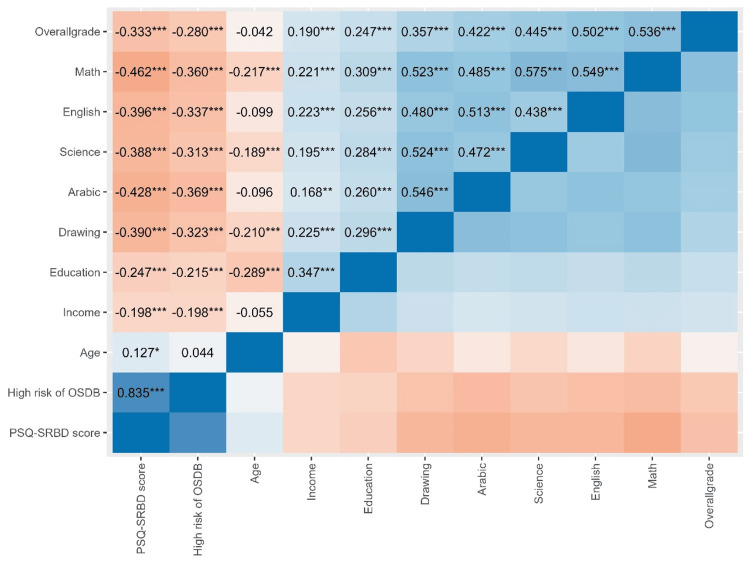
Correlation between the grades of the children and socio-demographic characteristics * p < 0.05, ** p < 0.01, *** p < 0.001 OSDB score: OSDB score as a continuous variable; OSDB: OSDB as yes/no, based on a cutoff of 0.33. OSDB, obstructive sleep-disordered breathing

High risk of OSDB was negatively associated with the grades of all subjects and with the overall grade (p < 0.001) (Figure 2).

**Figure 3:**
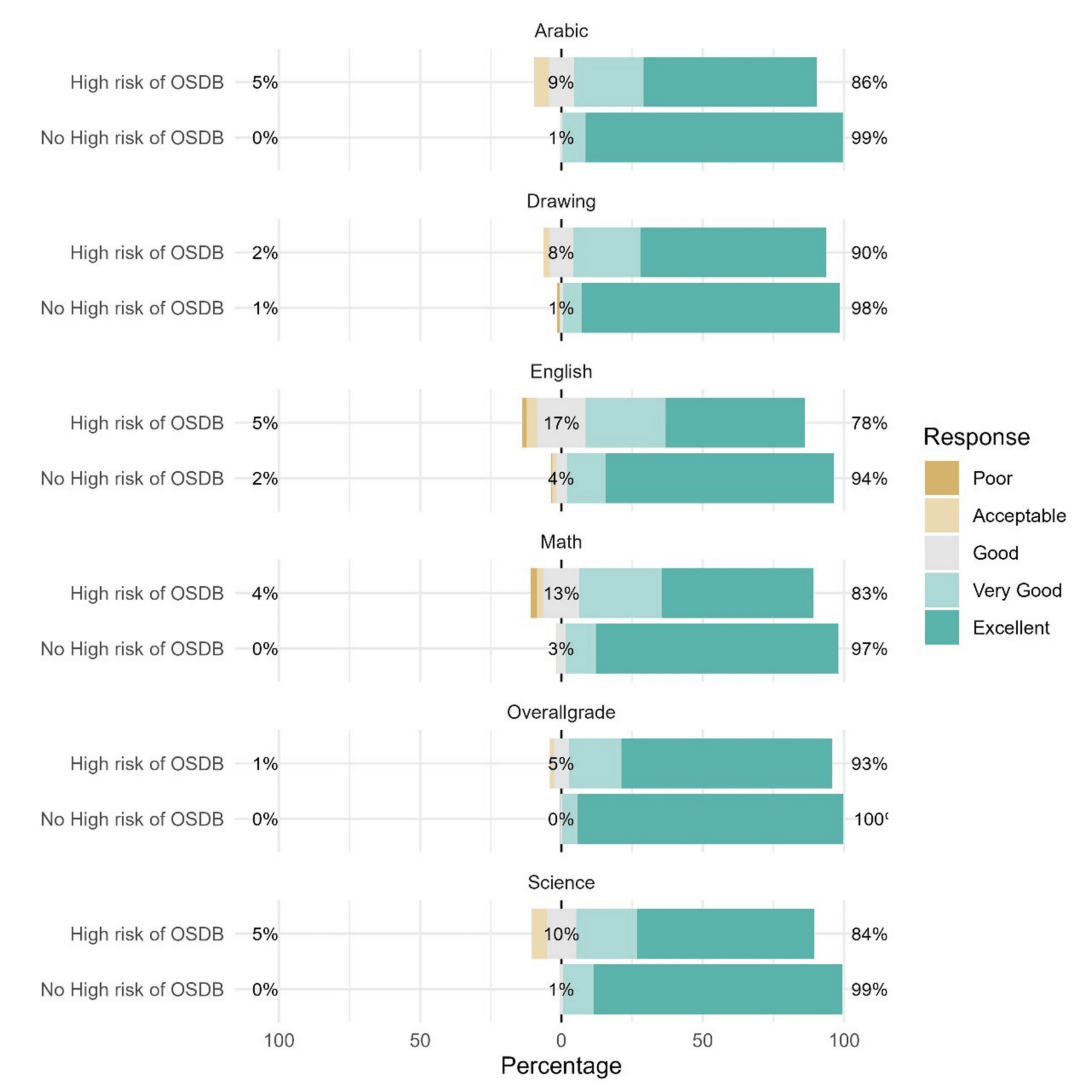
Association between high risk of obstructive sleep-disordered breathing (OSDB) and socio-demographic characteristics The numbers on the right represent the percentage of respondents who obtained excellent or very good grades. The numbers on the left represent the percentage of those who obtained acceptable or poor grades.

A comparison between children with and without high risk of OSDB is illustrated in Figure 3.

The high risk of OSDB score was negatively correlated with the grades of all participants and the overall grade (p < 0.001).

Results suggest that the distribution of grades was between respondents without OSDB.

The results showed that the average age was not significantly different between children with and without OSDB. Males were more prevalent among children with high risk of OSDB (85 (63.4%) vs. 106 (45.5%); p < 0.001). The distribution of school levels did not differ significantly between groups. Nasal symptoms were more severe in patients with high risk of OSDB. The prevalence of different comorbidities was significantly higher in children with high risk of OSDB (p < 0.001 for bronchial asthma) (Table 3).

**Table 3:** Factors associated with obstructive sleep-disordered breathing (OSDB) Categorical data were summarized using counts and percentages. Continuous variables were summarized using median (IQR). Categorical data were compared using the chi-square test of independence, and continuous data were compared using the Mann-Whitney U test.

	No ODSB	ODSB	Test statistics	Overall p-value
	N = 233	N = 134		
Age	10.0 (8.00; 13.0)	11.0 (8.00; 14.0)	8.48 (mean square)	0.395
Sex			10.97 (chi-square)	0.001
Male	106 (45.5%)	85 (63.4%)	-	-
Female	127 (54.5%)	49 (36.6%)	-	-
School level			1.498 (chi-square)	0.683
Primary elementary	93 (39.9%)	52 (38.8%)	-	-
Upper elementary	65 (27.9%)	32 (23.9%)	-	-
Intermediate	50 (21.5%)	31 (23.1%)	-	-
Secondary	25 (10.7%)	19 (14.2%)	-	-
Nasal condition				
Nasal obstruction	0.00 (0.00; 0.00)	0.50 (0.00; 3.00)	171.417 (mean square)	<0.001
Nasal discharge	0.00 (0.00; 1.00)	1.00 (0.00; 2.00)	88.319 (mean square)	<0.001
Recurrent sore throat	0.00 (0.00; 1.00)	2.00 (1.00; 3.00)	145.176 (mean square)	<0.001
Decreased hearing	0.00 (0.00; 0.00)	0.00 (0.00; 2.00)	73.492 (mean square)	<0.001
Hyponasality	0.00 (0.00; 0.00)	1.00 (0.00; 3.00)	208.378 (mean square)	<0.001
Chronic conditions				
Bronchial asthma	24 (10.3%)	39 (29.1%)	21.155 (chi-square)	<0.001
Cardiac	3 (1.29%)	3 (2.24%)	0.479 (chi-square)	0.673
Other chronic diseases	3 (1.29%)	9 (6.72%)	7.927 (chi-square)	0.011
No chronic disease	207 (88.8%)	85 (63.4%)	33.778 (chi-square)	<0.001
Total day + night sleeping hours			0.473 (chi-square)	0.563
<9 hours	106 (45.5%)	56 (41.8%)	-	-
>9 hours	127 (54.5%)	78 (58.2%)	-	-
Total night sleeping hours			0.025 (chi-square)	0.969
<9 hours	167 (71.7%)	95 (70.9%)	-	-
>9 hours	66 (28.3%)	39 (29.1%)	-	-
Child sleeping time			5.298 (chi-square)	0.029
Before 10 PM	65 (27.9%)	53 (39.6%)	-	-
After 10 PM	168 (72.1%)	81 (60.4%)	-	-
Monthly income			16.227 (chi-square)	0.001
<10,000 SAR	44 (18.9%)	44 (32.8%)	-	-
10,000-20,000 SAR	108 (46.4%)	66 (49.3%)	-	-
20,000-40,000 SAR	59 (25.3%)	15 (11.2%)	-	-
>40,000 SAR	22 (9.44%)	9 (6.72%)	-	-
Parent’s education			17.454 (chi-square)	<0.001
None has a bachelor’s degree	23 (9.87%)	30 (22.4%)	-	-
One has a bachelor’s degree or higher	65 (27.9%)	48 (35.8%)	-	-
Both have a bachelor’s degree or higher	145 (62.2%)	56 (41.8%)	-	-

Although sleeping hours were not significantly different between the groups, higher income levels were reported in parents of children without ODSB (p < 0.001).

Ordinal logistic regression was used to assess the association between high risk of ODSB and overall grade after adjusting for possible confounders. The dependent variable was the overall grade (1 to 5) and independent variables included school level, education level, risk of ODSB (high vs. low), monthly income, and gender. Education was included as an ordinal variable.

**Table 4:** Ordinal logistic regression analysis results

Predictors	Odds ratio	95% CI	p
Parent’s education level (one level increase)	1.74	1.08-2.82	0.023
Monthly income			
<10,000 SAR	Ref	-	-
10,000-20,000 SAR	2.28	1.04-5.00	0.038
20,000-40,000 SAR	2.57	0.75-8.82	0.134
Monthly income: >40,000 SAR	1.65	0.47-5.74	0.432
Risk of OSDB (high vs. low)	0.23	0.11-0.47	<0.001
Gender: female	0.98	0.50-1.92	0.945
School level			
Primary elementary	-	-	-
Upper elementary	0.76	0.33-1.75	0.520
Intermediate	1.00	0.42-2.39	0.992
Secondary	1.60	0.50-5.13	0.431

The ordinal logistic regression model identified several factors associated with grade outcomes. Parent’s education level showed a significant positive association with grade, with each one-level increase in education level being associated with 1.74 times higher odds of achieving a higher grade (95% CI: 1.08-2.82, p = 0.023). Compared to families earning less than 10,000 SAR, families with an income of 10,000-20,000 SAR had significantly higher odds of achieving a higher grade (OR = 2.28, 95% CI: 1.04-5.00, p = 0.038). However, income levels of 20,000-40,000 SAR (OR = 2.57, 95% CI: 0.75-8.82, p = 0.134) and above 40,000 SAR (OR = 1.65, 95% CI: 0.47-5.74, p = 0.432) were not significantly associated with grades.

The risk of OSDB had a significant inverse relationship with grade outcomes. High OSDB risk was associated with 77% lower odds of achieving a higher grade compared to low OSDB risk (OR = 0.23, 95% CI: 0.11-0.47, p < 0.001). Gender did not show a significant association with grade outcomes (OR = 0.98, 95% CI: 0.50-1.92, p = 0.945). Similarly, school level (upper elementary, intermediate, and secondary) showed no significant association with grade outcomes when compared to the primary elementary reference group.

**Figure 4:**
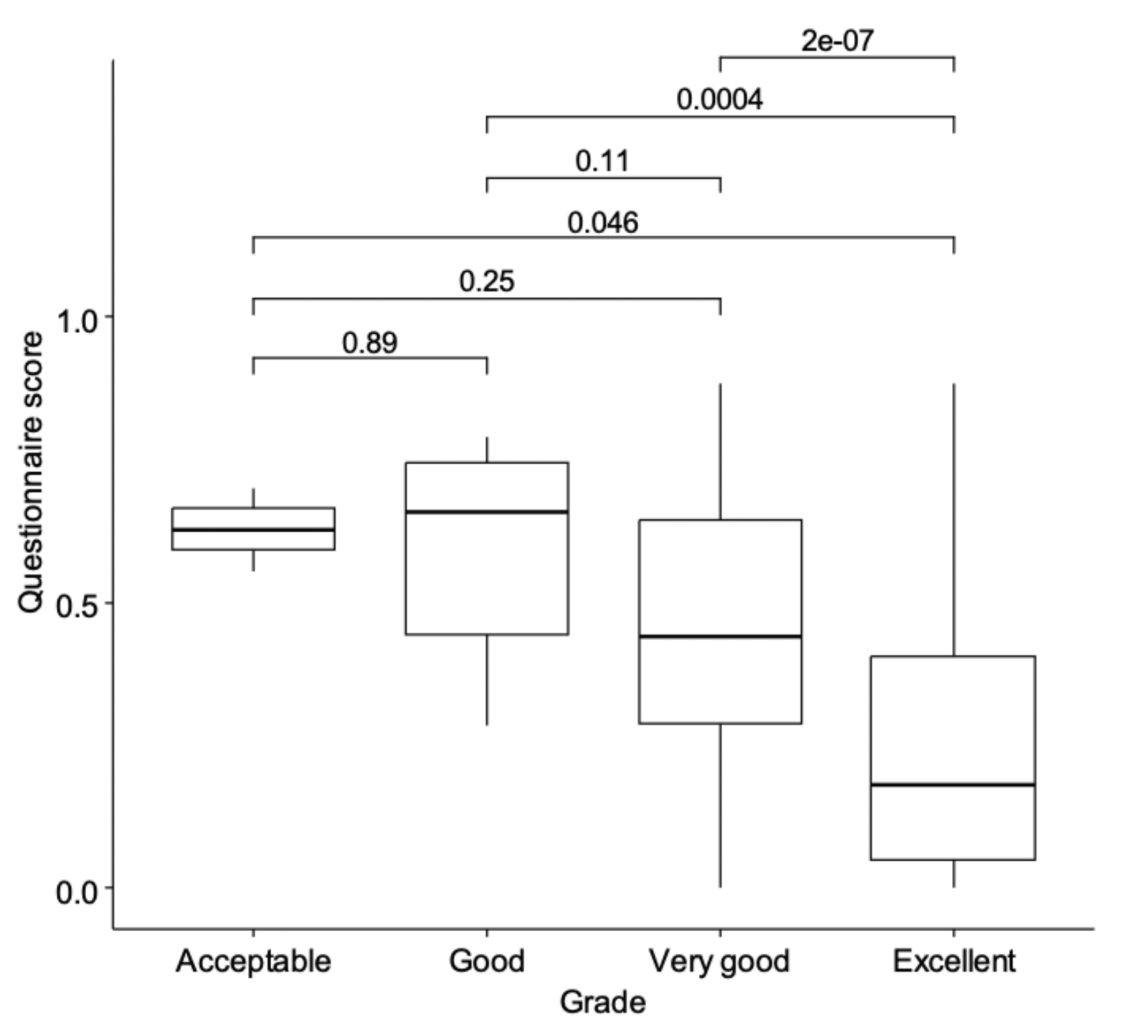
Comparison of paediatric sleep questionnaire-sleep-related breathing disorder (PSQ-SRBD) score between different grades

The overall grades were used for the analysis. The results of all the pairwise comparisons are presented in Figure 4.

The line in each box represents the median value. The upper and lower margins represent the 75 and 25% percentile, respectively. Pairwise comparisons using the Mann-Whitney U test showed a statistically significant difference in the distribution of high risk of OSDB scores between good and excellent grades, as well as between very good and excellent grades, with lower high risk of OSDB scores in children with higher grades. The Kruskal-Wallis test showed a statistically significant difference in the distribution of high risk of OSDB scores between the groups. The distribution of the high risk of OSDB scores also differed significantly between children with acceptable and excellent grades.

## Discussion

High risk of OSDB has garnered increasing attention due to its potential to adversely affect academic performance among school-aged children and adolescents. Therefore, we comprehensively explored the association between OSDB and academic performance, examining its various facets, from the prevalence of high risk of OSDB in this demographic to its consequences on educational attainment and risk factors contributing to its development. Our results underscore the critical role of educational institutions in recognizing and addressing the high risk of OSDB-related challenges to optimize the academic outcomes of affected students.

The findings of this study on the negative association between high risk of OSDB and academic performance are in line with extensive research on this topic. Beebe et al. found that children with untreated SDB demonstrated decline in cognitive and academic performance over time, confirming its detrimental impact on academics [13]. Furthermore, a comprehensive review by Trosman et al. reinforced this finding by revealing that children with high risk of OSDB had lower cognitive and academic test scores than those without [14]. O'Brien et al. investigated the academic performance of children with high risk of OSDB and reported that they scored lower levels in reading and math assessments [15]. Additionally, Archbold et al. conducted a longitudinal study that showed that children with severe OSDB experienced poorer academic performance over time, emphasizing the dose-response relationship between OSDB severity and academic outcomes [16].

This study also highlights the presence of comorbidities, such as bronchial asthma, in some children with OSDB, which aligns with the existing research. Kheirandish-Gozal et al. found a bidirectional association between OSDB and asthma in children [17]. Children with OSDB were more likely to have asthma and vice versa. In contrast, one study found no significant group differences in test scores when evaluating six separate executive function areas in a chosen sample of patients without comorbidities [18]. Therefore, they concluded that OSDB without comorbidities did not result in impaired executive function. However, Hilsendager et al. found that OSDB paired with obesity impairs executive function, and that obesity status is a strong predictor of performance on executive functioning tests [19]. 

This study emphasized the influence of parental education and income on academic outcomes, which is in line with the broader literature on socioeconomic factors and academic success. Duncan and Magnuson noted that children from higher socioeconomic backgrounds tended to perform better academically [20]. Sirin reviewed the impact of socioeconomic status on academic achievement and highlighted income and education as key factors shaping children's educational outcomes [21].

The current study found a higher prevalence of OSDB in males, consistent with the results of other studies. Li et al. reported a higher prevalence of OSDB in male children than in female children [22].

This study noted variations in sleep duration and bedtime patterns among children, which are a relevant aspect of this discussion. Owens et al. investigated the impact of irregular sleep schedules on academic achievement in adolescents and found that irregular sleep patterns were associated with poor academic performance, thus highlighting the importance of consistent sleep routines [23]. Moreover, Short et al. emphasized the significance of adequate sleep duration for academic success, noting that insufficient sleep can impair cognitive function and negatively affect educational outcomes [24].

Our results demonstrated significant differences in OSDB scores between different grade groups, with lower OSDB scores in children in higher grades. Mitchell et al. reported that greater OSDB severity was associated with lower academic performance, which is consistent with the results of the current study [25]. Biggs et al. assessed the impact of OSDB on neurocognitive function in children and found that children with more severe OSDB had poorer neurocognitive outcomes, including academic difficulties [26].

A longitudinal study by Rosen et al. examined the impact of OSDB on academic performance and found that children with untreated OSDB had lower academic achievements, and that academic improvement was associated with the resolution of OSDB [27]. Golan et al. examined SDB in school-aged children and found that children with OSDB had lower school achievement scores than those without [28]. Another study by Carotenuto et al. investigated the neuropsychological profiles of children with OSA and observed deficits in attention, memory, and executive function that could have contributed to academic difficulties [29]. Furthermore, Brockmann et al. examined the impact of OSA on school performance of children and found that children with severe OSA had lower grades and poorer school attendance than the controls [30].

A longitudinal study by Marcus et al. followed up children with OSDB after adenotonsillectomy and found improvements in academic performance and behavior [31]. Another study investigated academic achievement in children with OSDB and found that they had lower scores on standardized tests than children without OSA [32]. In addition, Friedman et al. assessed the academic performance of children with OSA and found that they had lower reading and math scores than controls [33]. Smith et al. examined the relationship between OSA and academic achievement and found that children with OSA had lower standardized test scores and poorer school attendance [34]. Molfese et al. investigated the effects of OSA on academic achievement and found that children with OSA had lower scores on tests of reading and math [35]. Jackman et al. examined the academic achievements of children with OSDB and found that they had lower scores on measures of reading, math, and overall academic achievement, although no cognitive-behavioural impairments were observed [36]. Giordani et al. assessed the neuropsychological functioning of children with OSA and found deficits in attention, memory, and executive functions that could contribute to academic problems [37].

Our study is limited by being an online questionnaire, so we did not use polysomnography or examine the patients clinically. Furthermore, the evaluation of academic performance is done based on grades rather than scores.

## Conclusions

In summary, the findings of this study showed that high risk of OSDB has been consistently associated with lower academic performance, underscoring the importance of early diagnosis and intervention to mitigate these effects. Further prospective studies are encouraged to clarify the long-term impact of high risk of OSDB and its treatment modalities on the academic and occupational performance of these patients.

## Appendices

**Table 5:** Questionnaire

Please fill this questionnaire for each school-age child.
First section: Socio-demographic data
Age (in numbers)
Gender
Male
Female
Weight in kg
Height in cm
School level
Primary school (first to third grades)
Primary school (fourth to sixth grades)
Middle school
High school
Does your child have any of these symptoms?
Nasal obstruction
Runny nose
Recurrent sore throat
Decreased hearing
Hypernasality
Others, please mention……...
No, he/she doesn’t have
Does your child have any of these diseases?
Bronchial asthma
Cardiac
Others, please mention……...
No, he/she doesn’t have
Has your child undergone any of these surgeries?
Adenoid/tonsils surgery
Tongue surgery
Palate surgery
Nasal surgery
Laryngeal surgery
Others, please mention……...
No, he/she hasn’t
Total sleep duration per day
≤9.00
>9.01
Sleeping duration per night
≤9.00
>9.01
When does your child sleep at night?
Before 10:00 pm
After 10:00 pm
Father's education
Illiterate
General education
Bachelor
Master/PhD
Mother's education
Illiterate
General education
Bachelor
Master/PhD
Family monthly income
<10,000 SAR
10,000-20,000 SAR
20,000-40,000 SAR
>40,000 SAR
Second section: Obstructive sleep-disordered breathing (OSDB)
While sleeping, does your child…
Snore more than half the time?
Yes
No
I don’t know
Always snore?
Yes
No
Snore loudly?
Yes
No
Have “heavy” or loud breathing?
Yes
No
Have trouble breathing, or struggle to breathe?
Yes
No
Have you ever seen your child stop breathing during the night?
Yes
No
Does your child tend to breathe through the mouth during sleep?
Yes
No
Does your child tend to breathe through the mouth during the day?
Yes
No
Does your child have a dry mouth when waking up in the morning?
Yes
No
Third section: Association of obstructive sleep apnea with school grades
Overall school grades
A
B
C
D
Mathematics
A
B
C
D
Arabic
A
B
C
D
Science
A
B
C
D
English
A
B
C
D
Drawing
A
B
C
D
